# Supplementary material for: Monitoring Stimulated Darkening from UV-C Light on Different Bean Genotypes by NMR Spectroscopy
Source: Molecules. 2022 Mar 23;27(7):2060. doi: 10.3390/molecules27072060 (PMC9000581; doi:10.3390/molecules27072060)
Supplement: Supplementary file 1 [file molecules-27-02060-s001.zip › molecules-1633418-supplementary.pdf]

Monitoring the stimulated darkening from UV-C light on different beans genotypes by NMR spectroscopy

Marilia V. Salvador[a], Flavio V. C. Kock[a], Isabella Laporte[b], Jean F. C. Paulino[b], Rodrigo S. Garcia[c], Luciana L. Benchimol-Reis[b], Luiz A. Colnago[c], Antonio G. Ferreira[a*]

[a]*Universidade Federal de São Carlos, São Carlos-SP, Brasil.*

[b]*Instituto Agronômico de Campinas, Campinas-SP, Brasil.*

[c]*Embrapa Instrumentação, São Carlos-SP, Brazil.*

*giba_04@yahoo.com.br

Table

Caption	Page
**Table S1.** Organic compounds identified in beans.	**S2**

Figure

Caption	Page
**Figure S1.** TD-NMR results obtained from (a) CPMG and (b) RK-ROSE signals for 'TAA DAMA', 'BRS ESTILO' and 'TAA PEROLA', genotype at zero time (initial color) (T0, red line), prolonged darkening after 90 days exposure to fluorescent light (12-hours photoperiod) (T90, green line) and accelerated darkening after 96 hours of exposure to UVC light (T96, blue line).	**S8**

Table S1. Organic compounds identified in beans.

**Metabolite and Structure**	**δ (^{1}H) (Multiplicity*, J (Hz))**	**δ (^{13}C)**
Aminoacids		
Arginine<br>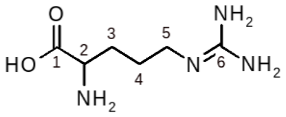	2- 3.79 (*m*)<br>3- 1.91 (*m*)<br>4-1.26 (*m*)<br>5- 3.24 (*o*)	2- 57.0<br>3- 30. 4<br>4- *no*<br>5- 43.3
Aspargine<br>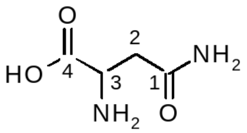	2- 2.88 (*m*)<br>3- 4.02 (*m*)	1-177.5<br>2- 37.3<br>3- 54.1<br>4- 177.3
Glycine<br>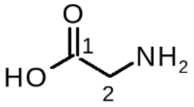	2- 3.20 (s)	2- 43.4
Isoleucine<br>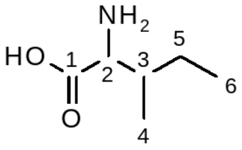	1-<br>2- 3.70 (*m*)<br>4- 1.00 (*d,* 7.02)<br>5- 1.20 (*m*)<br>6- 0.92 (*t*, 6.18)	1- 177.2<br>4-17.9<br>6-14.9
Leucine<br>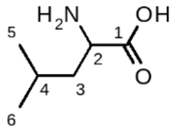	2- 3.40 (*o*)<br>3- 1.90 (*o*)<br>5,6- 0.94 (*d,* 6.27)	2- 43.6<br>3- 33.4<br>5,6- 23.9
Methionine<br>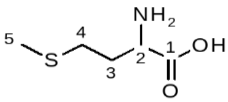	2- 3.78 (*o*)<br>3- 2.38 (*dd,* 7.54, 2.06)<br>4- 2.52 (*t,* 7.53)<br>5- 2.13 (*s*)	2- 57.2<br>3- 36.3<br>4- 34.6<br>5- 17.5

**s* – simplet; *d* – duplet; *t* – triplet; *dd* – double duplet; *m* – multiplet; o – overlapping signal; no – not observed.

Table S1. Organic compounds identified in beans (*Continue*).

Metabolite and Structure	δ (^{1}H) (Multiplicity*, J (Hz))	δ (^{13}C)
Aminoacids		
Phenylanine<br>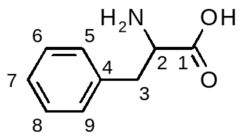	2- 4.05 (*o*)<br>3- 2.94 (*o*)<br>5,9- 7.34 (*d,* 6.96)<br>6,8- 7.43 (*t,* 7.42)<br>7- 7.37 (*m*)	2- 54.1<br>3- 37.3<br>5,9-130.0<br>6, 8- *no*<br>7-129.8
Serine<br>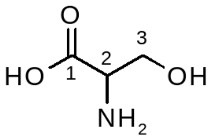	2- 3.77(*o*)<br>3- 4.07(*o*)	2- 57.2<br>3- 58.5
Threonine<br>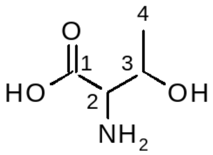	2- 3.60 (*m*)<br>3- 4.16 (*o*)<br>4- 1.34 (*d,* 6.73)	2- 61.9<br>3- 71.67<br>4- *no*
Tyrosine<br>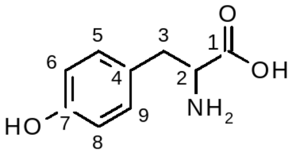	6,8- 6.91 (*m*)<br>5,9- 7.20 (*m*)<br>2- 3.90 (*o*)<br>3- 3.20 (*o*)	6,8- *no*<br>5.9- *no*

**s* – simplet; *d* – duplet; *t* – triplet; *dd* – double duplet; *m* – multiplet; o – overlapping signal; no – not observed.

Table S1. Organic compounds identified in beans *(continue)*.

**Metabolite and Structure**	**δ (^{1}H) (Multiplicity*, J (Hz))**	**δ (^{13}C)**
Aminoacids		
Tryptophan<br>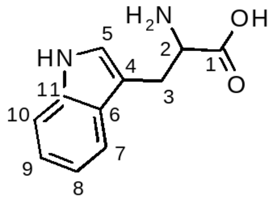	10- 7.39 (*m*)<br>9- 7.2 (*m*)<br>8- 7.88 (*d,* 7.99)<br>7- 7.74 (*m*)<br>5- 7.39 (*s*)<br>2- 4.09 (*m*)<br>3- 3.5 e 3.3 (*m*)	10- *no*<br>9-118.4<br>8- *no*<br>7-*no*<br>5-131.0<br>2- 58.5<br>3-*no*<br>1- 176.9
Valine<br>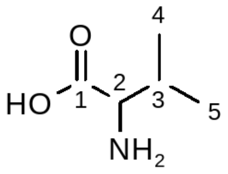	2- 3.6 (*o*)<br>3- 2.3 (*o*)<br>4- 1.05 (*d,* 7.04)<br>5- 1.004 (*d*, 7.03)	4-20.8<br>5-19.7<br>4- *no*<br>5- *no*
Carbohydrates		
Alpha-glucose<br>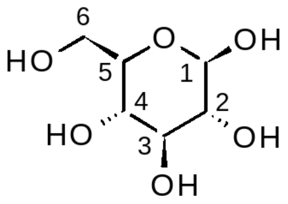	1- 5.23 (*d,* 3.83)<br>2- 3.47 (*m*)<br>3- 3.78 (*m*)<br>4- 3.51 (*m*)<br>5- 3.73 (*o*)<br>6-3.86 (*m*)	1- 95.0<br>2- 73.3<br>3- 75.7<br>4- 74.1<br>5- 63.3<br>6-75.7

**s* – simplet; *d* – duplet; *t* – triplet; *dd* – double duplet; *m* – multiplet; o – overlapping signal; no – not observed.

Table S1. Organic compounds identified in beans (*Continue*).

Metabolite and Structure	δ (^{1}H) (Multiplicity*, J (Hz))	δ (^{13}C)
Carbohydrates		
Beta-glucose 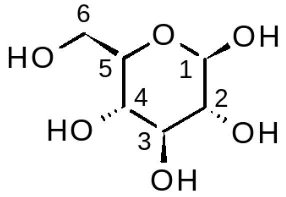	1- 4.65 (*d,* 8.03)<br>2- 3.49 (*o*)<br>3- 3.77 (*m*)<br>4- 3.54 (*m*)<br>5- 3.20 (*m*)<br>6- 3.76 (*m*)	1-98.8<br>2- 72.1<br>3- 75.4<br>4- 73.0<br>5- 56.7<br>6- 75.4
Xylose 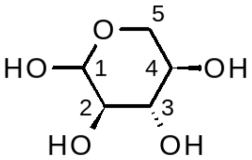	1- 4.59 (d, 7.87)<br>2, 4- 3.2 (*o*)<br>3- 4.17 (*o*)<br>5- 3.68 (*o*)	1- 99.3<br>5- 72.1<br>3- 71.7<br>5- 68.7
Sucrose 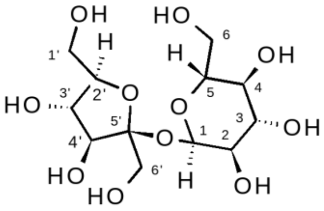	1- 5.42 (*d,* 3.86)<br>3'- 4.05 (*m*)<br>4'- 4.22 (*m*)	1- 95.0<br>3'- 76.8<br>4'- 76.2
Raffinose 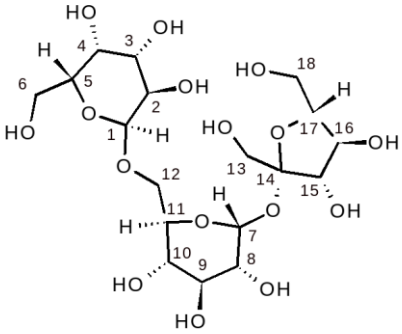	1- 5.02 (*m*)<br>7- 5.42 (*d,* 3.86)<br>15- 4.24 (*m*)	1- 101.0<br>7- 95.0<br>15- 79.2

s – simplet; *d* – duplet; *t* – triplet; *dd* – double duplet; *m* – multiplet; o – overlapping signal; no – not observed.

Table S1. Organic compounds identified in beans (*Continue*).

Metabolite and Structure	δ (^{1}H) (Multiplicity*, J (Hz))	δ (^{13}C)
Carbohydrates		
Stachyose 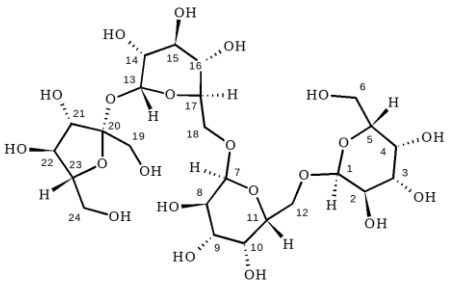	1- 5.00 (*m*)<br>7,13- 5.43 (*d*, 3.84)<br>21- 4.24 (*m*)	1- 101.0<br>7, 13 - 95.0<br>21-79.2
Verbascose 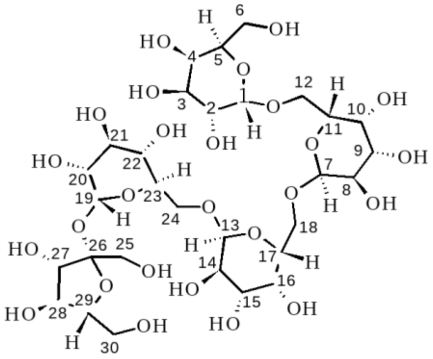	1- 5.02 (*m*)<br>7,13,19- 5.44 (*d*, 3.84)<br>3-4.24 (*m*)	1- 101.2<br>7- 95.0<br>3-79.2
Other compounds		
Trigonelline 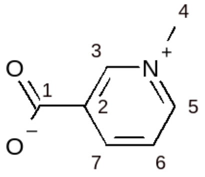	3- 9.13 (*s*)<br>4- 4.42 (*s*)<br>5, 7- 8.85 (*t,* 6.48)<br>6- 8.08 (*t,* 6.93)	3- 147.6<br>4- *no*<br>5,7- 148.6<br>6- 130.5<br>1-176.2

**s* – simplet; *d* – duplet; *t* – triplet; *dd* – double duplet; *m* – multiplet; o – overlapping signal; no – not observed.

Table S1. Organic compounds identified in beans (*Continue*).

**Metabolite and Structure**	**δ (^{1}H) (Multiplicity*, J (Hz))**	**δ (^{13}C)**
Other compounds		
Choline 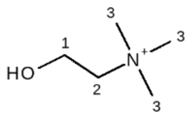	1- 4.08 (*m*)<br>2- 3.52 (*m*)<br>3- 3.19 (*s*)	1- 55.0<br>2- 56.5<br>3-57.0
Uracil 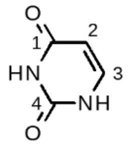	3- 7.55 (*d* 6.97)<br>2- 5.92 (*m*)	3- 106.5<br>2- *no*
<u>*Organic acids*</u>		
Fumarate 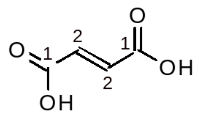	2- 6.52 (*s*)	*no*

*s – simplet; *d* – duplet; *t* – triplet; *dd* – double duplet; *m* – multiplet; o – overlapping signal; no – not observed.

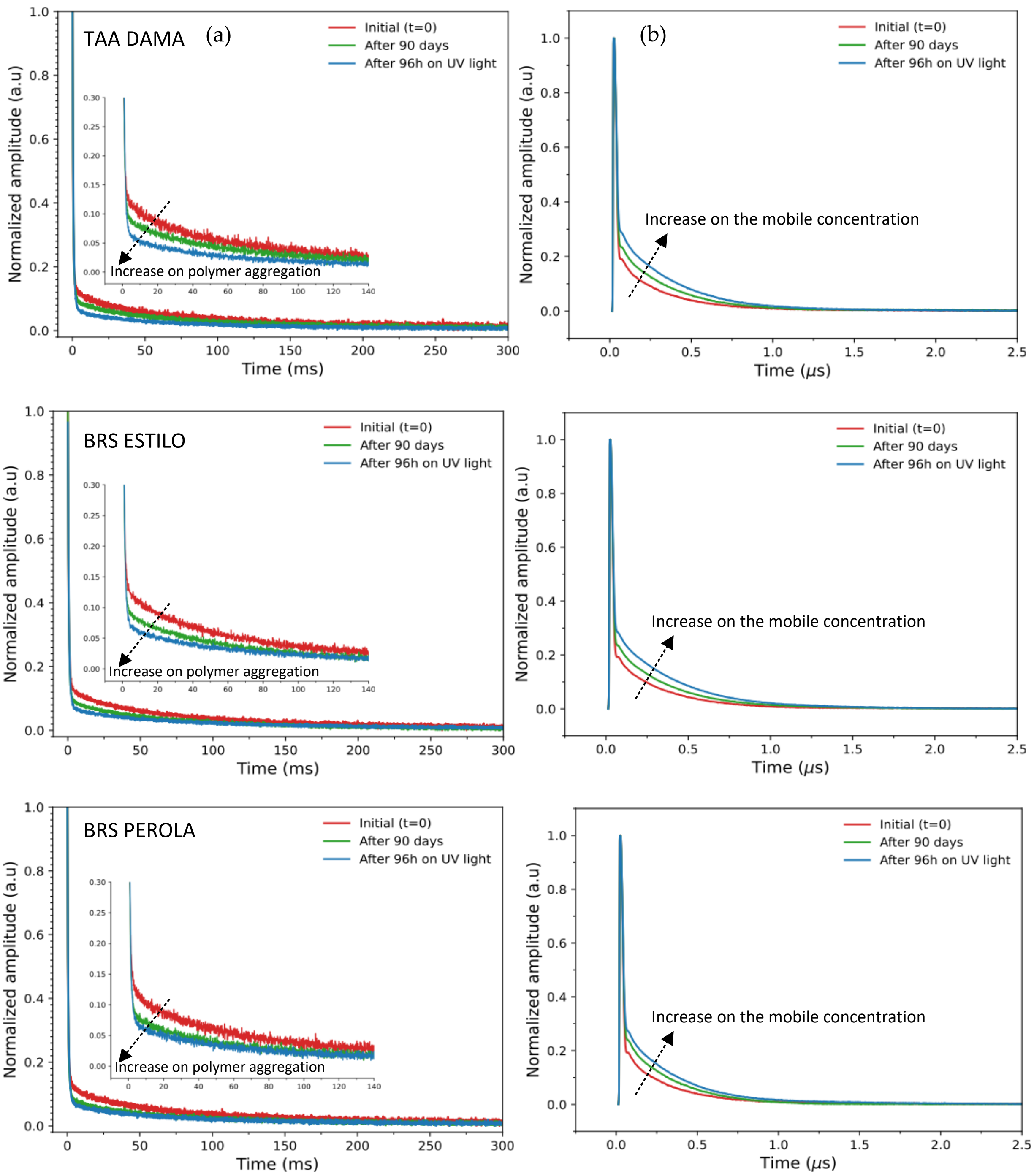

Figure S1. TD-NMR results obtained from (**a**) CPMG and (**b**) RK-ROSE signals for 'TAA DAMA', 'BRS ESTILO' and 'TAA PEROLA', genotype at zero time (initial color) (T0, red line), prolonged darkening after 90 days exposure to fluorescent light (12-hours photoperiod) (T90, green line) and accelerated darkening after 96 hours of exposure to UVC light (T96, blue line).
